# Editorial: The evolving role of lipid droplets: Advancements and future directions

**DOI:** 10.3389/fcell.2023.1175083

**Published:** 2023-03-21

**Authors:** Vineet Choudhary, Joel M. Goodman

**Affiliations:** ^1^ All India Institute of Medical Sciences (AIIMS), Department of Biotechnology, New Delhi, India; ^2^ Department of Pharmacology, University of Texas Southwestern Medical School, Dallas, TX, United States

**Keywords:** lipid droplets, organelle biogenesis, seipin, triacylglycerol, lipodystrophy, lipid storage disorders, neuronal lipid droplets, LD protein targeting

Lipid droplets (LDs), traditionally known as fat storage organelles in all cell types, have emerged as crucial players in diverse cellular functions, including protein trafficking and sequestration, endoplasmic reticulum (ER) stress responses, lipid trafficking, innate immunity, and assembly of viral particles [reviewed in (Welte and Gould, 2017; Olzmann and Carvalho, 2019)]. While LDs usually are key to maintaining lipid homeostasis, they can also cause severe pathologies including obesity, fatty liver, type-2 diabetes, atherosclerosis, neurological diseases, and cancer [reviewed in (Henne et al., 2019; Nagarajan et al., 2021; Islimye et al.)]. Although the biogenesis of LDs occurs in virtually all cell types, and huge progress has been recently made toward an understanding the mechanisms of this pathway, our knowledge is far from complete. Recent evidence suggests that LD biogenesis occurs at specialized subdomains of the ER network in a stepwise fashion. Many important LD assembly factors have been identified that orchestrate and ensure the fidelity of this process (Gao et al., 2019; Renne et al., 2020; Rao and Goodman, 2021; Schneiter and Choudhary, 2022). In this Research Topic, both original research and reviews highlight the novel insights into the dynamic functions of LDs and our current understanding of how alterations in LD homeostasis are implicated in disease pathogenesis.

LD biogenesis is driven by the accumulation of neutral lipids (NL), triglycerides (TAG) and steryl esters (SE), within the hydrophobic core of the ER membrane. Nascent LDs thereafter emerge from the ER toward the cytoplasm, enveloped in a phospholipid monolayer, and are covered by a specific set of LD-resident proteins (Walther et al., 2017; Chapman et al., 2019). Recent studies have revealed role of proteins, lipids, and biophysical properties that determine assembly of nascent LDs at discrete ER subdomains, a pre-requisite for maintaining ER homeostasis (Choudhary et al., 2020; Wang et al., 2018; Ben M’barek et al., 2017; Santinho et al., 2020). Seipin is one of the key proteins that determines sites where LD assembly machinery gets recruited to initiate phase separation of neutral lipids, and in doing so controls the number, size, and morphology of LDs (Chung et al., 2019; Choudhary et al., 2020; Zoni et al., 2021; Schneiter and Choudhary, 2022). Absence of seipin results in ectopic TAG packaging, leading to numerous tiny or few supersized LDs, many of which lack the full complement of LD surface proteins. Thus, an impaired adipogenesis results in abnormal distribution of body fat and insulin resistance.

In this Research Topic, Salo reviews the recent literature about the molecular function of seipin (Salo). He describes how the presence of seipin drives nucleation of TAG-lenses at a lower concentration in the ER than TAG alone, and hence, seipin defines the LD biogenesis sites. Lack of seipin results in accumulation of neutral lipids in the ER (Cartwright et al., 2015). Interestingly, recent cryo-EM structures of human, fly and yeast seipin revealed that it is comprised of a large membrane-embedded ring-shaped oligomer that is crucial for its function (Sui et al., 2018; Yan et al., 2018; Arlt et al., 2022). These structures have provided an insight into the role of different domains that allows nanoscale clustering of NLs thereby priming LD formation. Interestingly, mammalian and fly seipins contain hydrophobic helices (HH) within the luminal domain that are predicted to interact with the bilayer, where they can bind to NL. This domain, however, is absent in the yeast counterpart but may be provided by Ldb16, a seipin-binding partner (Klug et al., 2021; Arlt et al., 2022). Seipin associates with LDAF1, the likely homolog of yeast Ldo proteins, to form a stable complex that co-purifies with TAG thereby facilitating LD biogenesis *in vivo*. Molecular dynamic simulations suggests that seipin transmembrane domains contribute to clustering of TAG within the ER membrane (Kim et al., 2022). Seipin has been shown to be essential for the formation and maintenance of steryl ester-rich LDs (Cartwright et al., 2015; Renne et al., 2022). Moreover, the presence of seipin at multiple ER contact sites might play a vital role in the maintenance of these contacts and regulate trafficking occurring at these junctions (Salo).

Aberrant LDs due to the lack of seipin can have serious reproductive effects. Cao and coworkers provide evidence that loss of seipin/SEIP-1 in the oocyte of *C. elegans* results in embryogenesis defects and perturbation of eggshell formation. They report that a specific population of seipin-positive LDs is utilized after fertilization to form lipid-rich permeability barrier necessary for proper eggshell development. Surprisingly, the defect of the permeability and decrease of progenies in seipin-null worms were rescued by deleting LD coat protein, PLIN-1, and this rescue required the presence of RAB-18 (Cao et al.). The authors propose SEIP-1-dependent and -independent mechanisms involving LDs that act in parallel to control the packaging and export of lipid-rich permeability barrier constituents.

Besides eggshell formation in worms, trafficking of LDs is essential for milk production in mammals, where they are secreted by epithelial cells in the mammary gland. Prior to secretion, LDs bind to the apical plasma membrane to make LD-APM junctions, are enwrapped by the PM and then secreted (Heid and Keenan, 2005). The perilipin Plin2 is a part of the LD-APM junction, but its role in secretion has been unclear. Monks et al. now report that Plin2 is not required for milk droplet secretion (Monks et al.). However, it modulates all steps in this process, as shown in Plin2-knockout mice. In these animals the composition of the LD-APM is altered and its width (i.e., distance between the LD surface and plasma membrane) is shortened, the enwrapping step is prolonged, and secretion at the onset of lactation is sluggish.

The fat stored in all LDs, including those in the mammary gland, is principally esterified fatty acids, both saturated and unsaturated. Danielli et al. summarize an exploding field of the role of LDs in polyunsaturated fatty acid (PUFA) storage, release, and function (Danielli et al.). Storage of PUFAs in TAG and other esterified forms appears to be a double-edged sword. For example, it is widely believed that LDs protect cells from ferroptosis in part by sequestering PUFAs from lipid peroxidation, and this is certainly true in many cases, but in others, peroxidation may be promoted within the LD, making these organelles into time bombs. The review also points out that targeting LDs by drugs to release PUFAs and cause their oxidation and cell ferroptosis may be important for therapy of resistant cancers. Evidence for the emerging role of LDs in providing PUFA precursors of eicosanoids and docosanoids, essential signaling molecules, is also discussed.

The extent of NL storage in LDs reflects a balance of fat biosynthesis and mobilization. Lipid metabolism can be coordinated between cell types in a tissue. Growing evidence suggests that defects in lipid metabolism and its coordination among cells play a major role in neurodegeneration. Islimye and coworkers review the recent advances about the role of LDs in glial, neurons, and neural stem cells during health and disease (Islimye et al.). Lipid metabolism in human brain appears to be highly cell-type specific. Under physiological conditions, glia, ependymal cells, and microglia store LDs. Neurons contain few, if any, detectable LDs *in vivo,* however*,* because they actively turnover TAG, thus favoring lipolysis over biosynthesis under physiological conditions. In contrast, ectopic accumulation of LDs in neurons serve as a hallmark of several neurodegenerative diseases, ageing, and stresses including redox imbalance and lipotoxicity (Teixeira et al., 2021; Islimye et al.).

Proper lipid storage and mobilization depend on LD-associated proteins, the targeting of which has been intensively studied. Interestingly, the localization of proteins to LDs and peroxisomes, which can oxidize fatty acids, are linked. Pex19, a protein that is farnesylated, is well known for its ability to target membrane proteins to peroxisomes (Theodoulou et al., 2013). The contribution of Lyschik et al. now shows that Pex19 has an important function for LDs (Lyschik et al.). The authors generated HeLa cell lines where Pex19 is deleted or replaced by a C296S mutant preventing its farnesylation. They report that Pex19 farnesylation is not required for peroxisomal assembly or function. More interestingly, perhaps, is the finding that farnesylation is critical for a novel role of the protein, the targeting a subset of proteins to LDs. In an observation that could be related, farnesylation of Pex19 was also shown to be required to fully deplete LDs of TAG under catabolic conditions.

The original function of Pex19, seipin, and most other proteins involved in organelle assembly were discovered through forward genetics or proteomics. Sánchez-Álvarez et al. present a near-exhaustive review of progress made from unbiased approaches on the roles of LDs in several cell functions, including proteostasis, lipid metabolism, inter-organellar communication, and innate immunity. The authors start with comparing the advantages of several model systems and continue with genetic approaches (such as RNAi screens) with a visual (such as fluorescent microscopic) output. Next, the advantages of proteomic approaches are compared, including cutting edge interpretive methods such as hierarchical clustering of data. A review of methods that are coupled with perturbations of the system, such as starvation, ER stress, or exposure to bacterial lipoprotein is offered. Finally a thoughtful presentation of possible future directions of functional genomics and metabolic profiling to learn more about LDs concludes the review.

In summary, the present Research Topic of reviews and research articles highlight the recent advances in the field of LDs with novel insights into the mechanisms of LD assembly and function, and the ways in which disruption of LD homeostasis have important effects on diverse functions. However, several perplexing questions remain unanswered. Some revolve around sites of LD formation. A key question for subsequent studies is to determine how membrane geometry alters lipid and protein composition that promote LD formation. Seipin must play a role in this process, but many important details are not resolved. Does seipin traffic to preexisting specialized ER domains for LD formation (based on membrane lipid or protein composition), or is the domain defined by where seipin happens to be when it encounters NL diffusing through the membrane? What is the spatial relationship between sites of NL synthesis and LD assembly? Does seipin change its structure as LD bud from the bilayer? Ultimately, the role of seipin in preventing lipodystrophy is incompletely understood. For example, what are the relative contributions of seipin in adipocyte formation vs. maintenance?

Once LDs are formed, they are well known to associate with other organelles. Although several tethering proteins have been recently identified at these contacts, key issues have yet to be resolved. The dynamics of LD-mediated contact formation and release in response to environmental cues are poorly understood, as are the resulting changes in lipogenesis, lipolysis, and lipid transfer that often result. In addition, there may be other, presently unknown, communication modalities at these junctions. Acquiring a deep understanding of the biology of LD contacts will be essential to determining the impact that dysfunctional LD contact sites have in human pathologies such as neurological diseases. Work in the new few years will likely reveal much more of how sites for LDs are selected and the normal role of this organelle in intracellular communication and energy metabolism at levels of the cell, tissue, and whole organism, as well as more insight into pathological situations when these processes go awry. We are optimistic that with improved understanding of such normal and pathological mechanisms involving LDs, new therapeutic interventions will be developed to improve the health of individuals suffering from diseases of aberrant lipid storage and utilization.

## References

[B1] Arlt H. Sui X. Folger B. Adams C. Chen X. Remme R. (2022). Seipin forms a flexible cage at lipid droplet formation sites. Nat. Struct. Mol. Biol. 29, 194–202. 10.1038/s41594-021-00718-y 35210614 PMC8930772

[B2] Ben M'Barek K. Ajjaji D. Chorlay A. Vanni S. Foret L. Thiam A. R. (2017). ER membrane phospholipids and surface tension control cellular lipid droplet formation. Dev. Cell 41, 591–604 e7. 10.1016/j.devcel.2017.05.012 28579322

[B3] Cartwright B. R. Binns D. D. Hilton C. L. Han S. Gao Q. Goodman J. M. (2015). Seipin performs dissectible functions in promoting lipid droplet biogenesis and regulating droplet morphology. Mol. Biol. Cell 26, 726–739. 10.1091/mbc.E14-08-1303 25540432 PMC4325842

[B4] Chapman K. D. Aziz M. Dyer J. M. Mullen R. T. (2019). Mechanisms of lipid droplet biogenesis. Biochem. J. 476, 1929–1942. 10.1042/BCJ20180021 31289128

[B5] Choudhary V. El Atab O. Mizzon G. Prinz W. A. Schneiter R. (2020). Seipin and Nem1 establish discrete ER subdomains to initiate yeast lipid droplet biogenesis. J. Cell Biol. 219, e201910177. 10.1083/jcb.201910177 32349126 PMC7337503

[B6] Chung J. Wu X. Lambert T. J. Lai Z. W. Walther T. C. Farese R. V. JR. (2019). LDAF1 and seipin form a lipid droplet assembly complex. Dev. Cell 51, 551–563 e7. 10.1016/j.devcel.2019.10.006 31708432 PMC7235935

[B8] Gao M. Huang X. Song B. L. Yang H. (2019). The biogenesis of lipid droplets: Lipids take center stage. Prog. Lipid Res. 75, 100989. 10.1016/j.plipres.2019.100989 31351098

[B9] Heid H. W. Keenan T. W. (2005). Intracellular origin and secretion of milk fat globules. Eur. J. Cell Biol. 84, 245–258. 10.1016/j.ejcb.2004.12.002 15819405

[B10] Henne W. M. Reese M. L. Goodman J. M. (2019). The assembly of lipid droplets and their roles in challenged cells. EMBO J. 38, e101816. 10.15252/embj.2019101816 31028045 PMC6484397

[B11] Kim S. Chung J. Arlt H. Pak A. J. Farese R. V. J. Walther T. C. (2022). Seipin transmembrane segments critically function in triglyceride nucleation and lipid droplet budding from the membrane. Elife 11, e75808. 10.7554/eLife.75808 35583926 PMC9122495

[B12] Klug Y. A. Deme J. C. Corey R. A. Renne M. F. Stansfeld P. J. Lea S. M. (2021). Mechanism of lipid droplet formation by the yeast Sei1/Ldb16 Seipin complex. Nat. Commun. 12, 5892. 10.1038/s41467-021-26162-6 34625558 PMC8501077

[B14] Nagarajan S. R. Butler L. M. Hoy A. J. (2021). The diversity and breadth of cancer cell fatty acid metabolism. Cancer Metab. 9, 2. 10.1186/s40170-020-00237-2 33413672 PMC7791669

[B15] Olzmann J. A. Carvalho P. (2019). Dynamics and functions of lipid droplets. Nat. Rev. Mol. Cell Biol. 20, 137–155. 10.1038/s41580-018-0085-z 30523332 PMC6746329

[B16] Rao M. J. Goodman J. M. (2021). Seipin: Harvesting fat and keeping adipocytes healthy. Trends Cell Biol. 31, 912–923. 10.1016/j.tcb.2021.06.003 34215489 PMC8526392

[B17] Renne M. F. Corey R. A. Ferreira J. V. Stansfeld P. J. Carvalho P. (2022). Seipin concentrates distinct neutral lipids via interactions with their acyl chain carboxyl esters. J. Cell Biol. 221, e202112068. 10.1083/jcb.202112068 35938957 PMC9365673

[B18] Renne M. F. Klug Y. A. Carvalho P. (2020). Lipid droplet biogenesis: A mystery "unmixing. Semin. Cell Dev. Biol. 108, 14–23. 10.1016/j.semcdb.2020.03.001 32192830

[B21] Santinho A. Salo V. T. Chorlay A. Li S. Zhou X. Omrane M. (2020). Membrane curvature catalyzes lipid droplet assembly. Curr. Biol. 30, 2481–2494 e6. 10.1016/j.cub.2020.04.066 32442467

[B22] Schneiter R. Choudhary V. (2022). Seipin collaborates with the ER membrane to control the sites of lipid droplet formation. Curr. Opin. Cell Biol. 75, 102070. 10.1016/j.ceb.2022.02.004 35306312 PMC7615794

[B23] Sui X. Arlt H. Brock K. P. Lai Z. W. Dimaio F. Marks D. S. (2018). Cryo-electron microscopy structure of the lipid droplet-formation protein seipin. J. Cell Biol. 217, 4080–4091. 10.1083/jcb.201809067 30327422 PMC6279392

[B24] Teixeira V. Maciel P. Costa V. (2021). Leading the way in the nervous system: Lipid Droplets as new players in health and disease. Biochim. Biophys. Acta Mol. Cell Biol. Lipids 1866, 158820. 10.1016/j.bbalip.2020.158820 33010453

[B25] Theodoulou F. L. Bernhardt K. Linka N. Baker A. (2013). Peroxisome membrane proteins: Multiple trafficking routes and multiple functions? Biochem. J. 451, 345–352. 10.1042/BJ20130078 23581405

[B26] Walther T. C. Chung J. Farese R. V. JR. (2017). Lipid droplet biogenesis. Annu. Rev. Cell Dev. Biol. 33, 491–510. 10.1146/annurev-cellbio-100616-060608 28793795 PMC6986389

[B27] Wang S. Idrissi F. Z. Hermansson M. Grippa A. Ejsing C. S. Carvalho P. (2018). Seipin and the membrane-shaping protein Pex30 cooperate in organelle budding from the endoplasmic reticulum. Nat. Commun. 9, 2939. 10.1038/s41467-018-05278-2 30054465 PMC6063905

[B28] Welte M. A. Gould A. P. (2017). Lipid droplet functions beyond energy storage. Biochim. Biophys. Acta Mol. Cell Biol. Lipids, 1862, 1260–1272. 10.1016/j.bbalip.2017.07.006 28735096 PMC5595650

[B29] Yan R. Qian H. Lukmantara I. Gao M. Du X. Yan N. (2018). Human SEIPIN binds anionic phospholipids. Dev. Cell 47, 248–256 e4. 10.1016/j.devcel.2018.09.010 30293840

[B30] Zoni V. Khaddaj R. Lukmantara I. Shinoda W. Yang H. Schneiter R. (2021). Seipin accumulates and traps diacylglycerols and triglycerides in its ring-like structure. Proc. Natl. Acad. Sci. U. S. A. 118, e2017205118. 10.1073/pnas.2017205118 33674387 PMC7958289

